# Effects of seawater sulfur starvation and enrichment on *Gracilaria gracilis* growth and biochemical composition

**DOI:** 10.1038/s41598-022-15303-6

**Published:** 2022-06-30

**Authors:** Fethi Mensi, Aziz Ben Ghedifa, Hayfa Rajhi

**Affiliations:** Institut National des Sciences et Technologies de la Mer-Centre Kheiredine, 29 Rue Général Kheiredine, 2015 Le Kram, Tunisie

**Keywords:** Biogeochemistry, Ecology, Ocean sciences

## Abstract

The genus Gracilaria, largest biomass producer in coastal regions, encompasses a wide range of species including *Gracilaria gracilis*. Nowadays, there is a spate of interest in its culture in lagoon where the water sulfate concentration is variable. A laboratory culture was carried out to determine the sulfate concentration effect on their growth as well as their biochemical composition, which were 2.5, 27 or 50 mM, referred to as SSS (sulfur starved seawater), SW (seawater) and SES (sulfur enriched seawater).We found that the sulfate content of the surrounding medium is a key parameter influencing both the alga growth and its composition. However, seawater proved to be the most suitable environment to sustain alga growth, proteins, R-phycoerythrin and agar yields, but sulfur enrichment and starvation affects them. The sulfate degree of agar and therefore its quality is related to the medium sulfate concentration. We conclude that sulfur starvation (2.5 mM) for three weeks, led to severe growth retardation, lower agar yield and quality and indicated the limit potential of *G. gracilis* for mariculture under these conditions. These results demonstrated that the success of *G. gracilis* culture in the lagoon is feasible if sulfate concentration is closer to that of seawater.

## Introduction

Sulfur is an essential element for proteins, lipids and various metabolites. It contributes to the binding of metal ions and proteins, which is intrinsic for that all organisms to grow and survive, including algae. However, algae are known to synthesize and store more sulfur compounds than terrestrial plants^1^. After assimilation at lower oxidation state, sulfur occurs in the proteins through the amino acids cysteine, methionine and various other sulfur compounds, which are essential for cell growth, and whose synthesis is regulated by the sulfur metabolism network^2,3^. Nevertheless, at higher oxidation states, it occur in lipids, cell walls and photosynthetic membranes^4,5^. In aquatic environments, sulfur compounds have two interdependent transformation pathways. The first corresponds to the incorporation of sulfur into organic matter. However, the sulfur incorporated in aquatic organisms does not exceed 2% of their dry weight^6^. The second way in which it is used as an acceptor or donor of electrons represents an exclusively bacterial sulfur cycle, which takes place within the cell substance of the biota of the oceans, seas, and coastal environments^7^. Coastal environments represent 2.7% of the open sea area and contains lagoons presenting a socio-economic interest. Indeed, the main economic sectors of lagoon are fishing and shellfish harvesting^8^ and recently algae culture mainly *Gracilaria gracilis*.

The red alga *Gracilaria* has been identified as a potential species for aquaculture in Lagoon, where many attempts to develop their culture were performed in the Bizerte Lagoon (North Tunisia) and the Nador Lagoon (North Morocco). However, in the lagoon *G. gracilis* under wentstrong seasonal and area fluctuations in sulfates concentrations, which vary from 2.5 mM to 50 mM in Bizerte Lagoon^9^. The seaweeds need to be native to the location of aquaculture. However, according to^10^, Gracilaria has been basically cultivated in China (70%) and Indonesia (28%) in near-shore. It can be developed in estuarine and near-coast locations^11^. Hence, sulfate may not be a limiting factor for their growth and productivity in this area, which could explain the scarcity of data in literature on the topic compared to other factors; essentially nitrogen and phosphorus. However, information related to sulfates effect on *G. gracilis* growth and biochemical composition has not yet been reported. Accordingly, examining the effects of various factors (biotic and abiotic) on algal growth and biochemical composition provides us with a baseline for determining when a specific nutrient might become limiting. From this perspective, it seems of great importance to investigate sulfur effect on the growth and biochemical composition of *G. Gracilis* because how this alga adapts to sulfur deprivation is not well studied but is well explored in microalgae (green and red) as asserted by^12^. Over the last two decades, research in microalgae field has thrived referring to the biotechnological applications for the production of biofuels (bioethanol from starch and biodiesel from lipids) and bio-oil^13,14^. Nitrogen, phosphorus and sulfur starvation were the most widely used approaches for enhancing both starch and lipid accumulation^15^. In contrast, in red seaweeds culture we are rather interested in avoiding nutrients depilation and their consequence on alga growth and sulfated polysaccharides quantities and qualities.

Sulfated polysaccharides commercial value depend on the yield and gel quality, which is related to the negative effect of starch and different extraction techniques^16,17^. The starch content seems to depend on the stress that the algae experienced. As a matter of fact, the effects of environmental factors (light, nutrient supply, salinity, and temperature) on the content of starch and agar have been studied. The effects of nitrogen, and how other environmental factors interact with itin affecting agar quality have been explored^17,18^. So far, a few studies have investigated the effect of sulfur deprivation^19,20^. According to^17^, sulfate availability does not have immediate (< 5 days) effects on agar quality, but a long-term treatment, is necessary to better and deeper understand the relationship between sulfate availability and agar quality. State of arts works on the effect of sulfur starvation in seaweeds proteins and pigments content are rather limited compared to nitrogen, which is well addressed in literature. According to^21,22^ a loss of soluble protein and chlorophyll in microalgae is reported after sulfate starvation but prouved to be lower than those of nitrogen or phosphorus. Nitrogen, sulfur or phosphorus limitation reduces phycobiliprotein synthesis and results in their degradation (differentially affected by nutrient starvation), leading to a dramatic bleaching phenomenon known as chlorosis^23,24^.

The main objective of the present study, which represents to our knowledge the first report on the issue, resides in studying the effects of sulfur starvation and enrichment on the red alga *G. gracilis* growth and its biochemical composition. We also aim, to gather information regarding the possibility of its future culture in the lagoon.

## Material and methods

### Treatments and culture medium preparation

Three treatments such as, (I) the sulfur starved seawater (SSS), (II) the seawater (SW) and(III) the sulfur enriched seawater (SES) contain a sulfates concentration of 5 mM, 27 mM and 50 mM respectively, were used to *Gracilaria gracilis* algae culture*;* the only difference between all treatments was the sulfates concentration. The natural seawater was sampled in April 2019, from Kheireddine Shore (36°49′30.4″, 10°18′57.9″), near the National Institute of Marine Sciences and Technologies in the North of Tunisia. The pumped Sea water was firstly filtered according to^25^, secondly was sterilized, by adding a 1 mL of commercial bleach per one liter of seawater. A carefully mixture was realized, then, the water stands for several hours in the dark conditions. The water being treated, was neutralized with a sodium thiosulfate (Na_2_S_2_O_3_.5H_2_O) solution of 250 g L^−1^, 1 mL of the resulted mixture was added for each 4 mL of bleach used^26^. Finally, a sufficient quantity of prepared seawater (PS) was obtained and used to prepare the treatments (SSS, SW and SES) and the culture medium (CM).

#### Von Stosch (Grund) Medium from^27^ preparation

A modified Von Stosch (Grund) medium according to^27^ was prepared. This medium is suitable for diverse red types of seaweed culture^28^. In order to reduce the bacterial growth, the Tris buffer that basically used in Grund Medium was removed from the following protocol. Then, a 940 ml of prepared seawater (PS) was pasteurized and 10 ml of each stock solution were added aseptically. The medium was subsequently autoclaved. All constituents and vitamins, used to prepare stock solution, were indicated by^25^.

#### Sulfur starved seawater treatment (SSS)

The sulfates ions were added to BaCl_2_ to obtain BaSO_4_ according to the following equation:

$${\text{Ba}}^{{{2} + }} \;\left( {{\text{aquous}}} \right) + {\text{SO}}_{{4}}^{{{2} - }} \;\left( {{\text{aquous}}} \right) \to {\text{BaSO}}_{{4}} \;\left( {{\text{solid}}} \right)$$

The Barium Sulfate (BaSO_4_) has very low solubility in water (Ksp = 1.1 × 10^−10.1^). The concentration of sulfate ions in seawater were calculated by plotting the conductance of the solution as a function of the volume of the reagent. A BaCl_2_ solution (C = 5.0 × 10^–2^ M) was used to titrate a 50 mL of seawater prepared in a 500 mL beaker. The data was then graphed using MS Excel and splitting the data into two segments at the minimum conductivity and obtaining a trend line for each segment. The end point was determined by setting the two trend line equations equal to each other and solving for the sulfate concentration (unknown variable), which was determined graphically by the two lines intersecting. A calibration range of BaSO_4_ concentrations were used to seawater sulfate precipitation until to obtain a seawater sulfate free. A 40 L of previously prepared seawater (placed in aquarium with a volume of 50 L) and 1 L of the barium chloride solution were applied to obtain a finally sulfates concentration of 2.5 mM, corresponding to the sulfur starved seawater treatment (SSS).

#### Sulfur enriched seawater treatment (SES)

A3.26 g of Na_2_SO_4_ was added to 1 L of prepared seawater (PS) to obtain a sulfate concentration of 50 mM, corresponding to sulfur enriched seawater treatment (SES).

#### Seawater treatment (SW)

A prepared seawater (PS) obtained as indicated above, which contain a sulfates’ concentration of 27 mM, was used as the seawater treatment (SW).

### Seawater sulfate determination

Seawater Sulfate content was determined as described by^29^ with modification. The conditioning reagent contains 150 g NaCl, 100 ml glycerol (126 g), 60 ml concentrated HCl and 200 ml of ethanol (95%). These different reagents were mixture in 1 L of deionized water. A 1 ml of conditioning reagent and the sample (1 mL) were thoroughly mixed. A 60 mg of crushed barium chloride was added and mixed for 30 s. The mixture was immediately measured at the absorbance of 420 nm. A mixture excluding sulfate was used as blank. A standard curve ranged from 0 to 0.25 mM was used to determine the different corresponding absorbance concentration. The sample should be diluted and adjusted into the range standard curve (0 to 0.25 mM), that would be the main modification of^29^ method.

### Preparation of biological materials and experiments

The red alga *Gracilaria gracilis* was collected in March 2019 from the Bizerte Lagoon (37°22′32.6; 9°91′62.8) in North Tunisia. The site collection was characterized by a salinity of 36 psu, a temperature of 15 °C and a low transparency of the water. The samples are transported to the laboratory in a cool box containing seawater, where they are cultured in a large tank of water with a salinity of 36 psu and a temperature of 15 °C without the addition of nutrients. This experimental step was carried out in order to deplete the nutrients stores within the algae and obtain homogenous thalli according to^30^. The algae with bright-red thalli were distributed in three aquariums. Indeed, the thalli with the same color, at the same stage of the development cycle and those having similar lengths and branches (homogeneous) were chosen. Subsequently, these thalli were transferred in aerated tanks containing filtered seawater, illuminated with white light fluorescent lamps (Philips Actinic BL, TL 15 W, Poland) at 7 µmol photons m^−2^ s^−1^, under a 12:12 light: dark photoperiod, temperature and salinity were maintained at 15 °C and 36 psu, respectively. During the pre-culture period (one-week), the algae remained healthy. The aim of this step is to deplete the algae nutrient stocks and homogeneous thalli proliferation^31^. The selected thalliof 100 ± 2 g were randomly distributed in a 30L glass aquarium filled with seawater (SW), sulfur starved seawater (SSS) and sulfur enriched seawater (SES), with stocking density of 5 g fw L^−1^^18^ for three weeks (experimental period). The treatment was carried out under a 12:12 light:dark photoperiod in the laboratory. However, the environmental parameters such as light (180 µmol photons m^−2^ s^−1^) water temperature (20 °C), pH (8), salinity (36 psu), ammonium (80 μmol g^−1^ fw L^−1^) and nitrate (80 μmol g^−1^ fw L^−1^) were kept constant throughout the experiment as indicated by^18^. Seawater was exchanged and nutrients renewed every three days. In addition, every day a 1 mL of vonStosch (Grund) Medium was used for all treatments and we take into account the nitrate that contain, which is equal to 5 µM mL^−1^ when we add nitrate quantity. Five replicates per treatment were used. Light was measured by LICOR data logger (LI 1000). Salinity was maintained at 36 psu by distilled water and Nacl. The cultures were well mixed by air-bubbling. Stock solutions were prepared before each medium renewal. Phosphate (KH_2_PO_4_) solution was prepared, with a sufficient amount at the start-up of the experiment. The Phosphate was added in the different treatments to avoid their limitation. However, the molar N:P ratio was maintained through the experimental period equal to 10:1. The Germanium dioxide (1 mL L^−1^) was added to the cultures to inhibit the diatom growth^32^.

The used glass material was washed with 10% of HCL and deionizer water to prevent possible nutrient contamination. The water in the flasks was aerated to ensure water motion. Water was changed twice weekly to avoid CO_2_ and nutrient limitation risk. For every medium change, throughout the culture period, fresh weight (fw) biomass was weighted and added solution was adjusted according to the obtained weight.

### Daily growth rate

The weights were recorded at the start-up g and at the end of the experiments in order to calculate the daily growth rate (DGR). The calculated daily growth rate (DGR), was expressed as the percent (%) increase in thalli weight per day, according to^33^ formula: DGR (% day^−1^) = In (Wf/W0)/t × 100. Wf is the final weight after the t (days) culture, and W0 is the initial fresh weight**.**

### Agar extraction and characterization

At the end of the culture period, firstly the dry and cleaned seaweed samples were washed with tap water. Secondly, they were placed in 400 mL of 5% H_2_SO_4_ solution during 1 h at room temperature (25 °C). Then, were further rinsed thoroughly with tap water. Finally, the agar extraction was performed in glass bottles (500 mL) at 100 °C during 80 min using an alga to water ratio of 3:100 (w/v).

The heated solution was mixed with diatomaceous earth and filtered with pressure. The obtained filtrate was transferred to a flat steeled recipient until it was cooled at room temperature lasting 15 to 20 min. The supernatant was stocked in at − 18 °C overnight. The next day, the filtrate was thawed at room temperature until a thin agar film was formed. The agar yield was calculated as the following formula:

$$Agaryield(\mathrm{\%})=\left(Wa/Ws\right)\times 100$$

where *W*_*a*_ is the dry agar weight and *W*_*s*_ is the dry seaweed weight (g).

Sulfate content in polysaccharides was determined by the barium chloride-gelatin^25^. A standard curve was made as following: 0.02; 0.04; 0.06; 0.08; 0.10; 0.12; 0.16; 0.18 and 0.20 mL of K_2_SO_4._ The standard solution (0.6 mg mL^−1^) were dispensed into test tubes; hydrochloric acid (1 M) was compensated to 0.2 mL solution. Then, a 3.8 mL of trichloroacetic acid (3% v/v) and 1.0 mL of barium chloride-gelatin solution (5 g L^−1^) were added and mixed. The absorbances were measured at 360 nm after incubation time lasting 15 min at room temperature. A 0.2 mL of hydrochloric acid solution was used as a blank. The 3,6-AG content was determined by the colorimetric method of^34^, using the resorcinol–acetal reagent and with fructose as standard. An agar solution (0.02 mg mL^−1^) of 2 mL was transferred into 20 × 150 mm screw cap tube, containing 10 mL of cold resorcinol-acetal reagent. The mixtures were mixed thoroughly and allowed to cool in an ice bath during 3 min. The tubes were placed in 20 °C water bath lasting 4 min and then heated at 80 °C during 10 min in the dark. After cooling, the absorbance at 555 nm was read within 15 min. Commercial agar was used as a reference. A standard curve was prepared using D-fructose at concentration ranging from 0.018 to 0.09 mM. The 3,6-AG content was calculated and expressed as the percentage (dry weight basis) of agar. Experiments were performed, in triplicate (n = 3).

### Proteins, R-phycoerythrin (RPE) and total soluble carbohydrates determination

The total soluble carbohydrate, proteins and R-phycoerythrin extraction and contents were determined as described by^35^. The total soluble sugars were determined in the supernatant according to^36^. Crude protein was determined according to the method of^37^.

### Statistical analysis

All results are expressed as mean ± SD. After verification of the homogeneity of the variances and the normality of the data, the results relative to all studied responses were submitted to one way ANOVA analysis according to the procedure GLM of the Statistica software version 5.1 (Statsoft, Tulsa, USA). When ANOVA proved to be significant, the Duncan test was used to compare averages; the significance level of 5% is retained.

## Results

**Figure 1:**
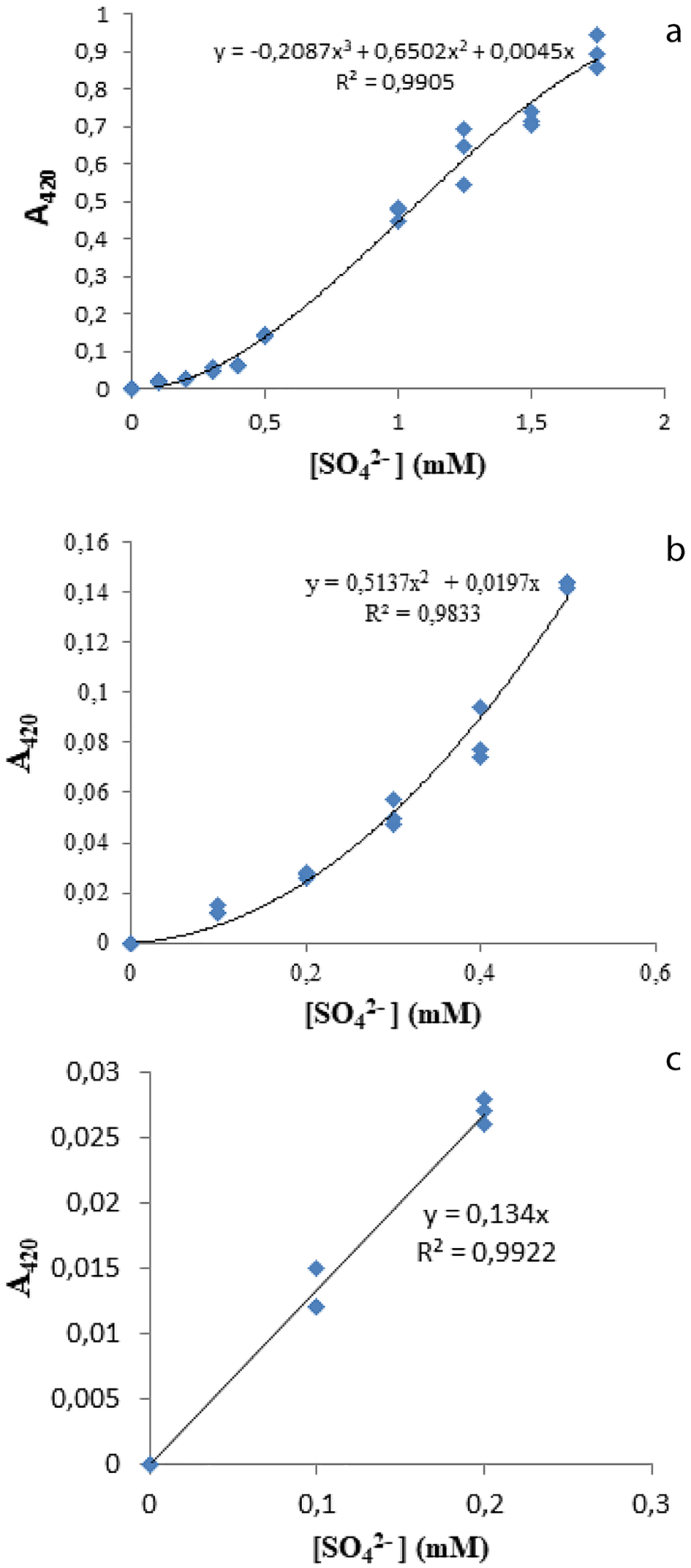
**(a)** Standard curves fitted with a third-order polynomial, **(b)** second order polynomial and **(c)** linear. These curves are described by their equations and their correlation values (R^2^), where y is the absorbance at 420 nm (A_420_) of a sample and x is the corresponding sulfate concentration [SO_4_^2−^]in mM.

The old gravimetric procedures for the determination of sulfate in water, are insensitive and tedious, have been superseded. The methods currently opted for are based on the precipitation of sulfate with barium ions and are followed by turbidimetric or nephelometric monitoring of the resulting suspension^38^. A standard turbidimetric assay for the determination of sulfate concentration in water (range between 0 and 5 mM) was modified in order to achieve a faster and simpler method to determine sulfate in *G. gracilis* culture. A linear relationship between optical density and concentration is recorded for sulfate between 0 and 0.2 mM.The relationship became nonlinear at a higher concentration of sulfate (> 0.2 mM); a second-order between 0 and 0.6 mM and third-order polynomial between 0 and 2 mM with a regression coefficient of 0.98 for all curves (Fig. 1). When sulfate concentrations are higher than 0.2 mM, attempts to linearize the data have resulted in a poor fit. Furthermore, these models did not match the data between 0.6 and 2 mM, which requires the use of a third-order polynomial model. In analytical chemistry, we invest the calibration curve equation to calculate the concentration of the unknown sample. In the marine environment, the sulfate concentration is generally higher. In order to determine these concentrations, which are higher than 0.2 mM, dilutions need to be carried out.

**Figure 2:**
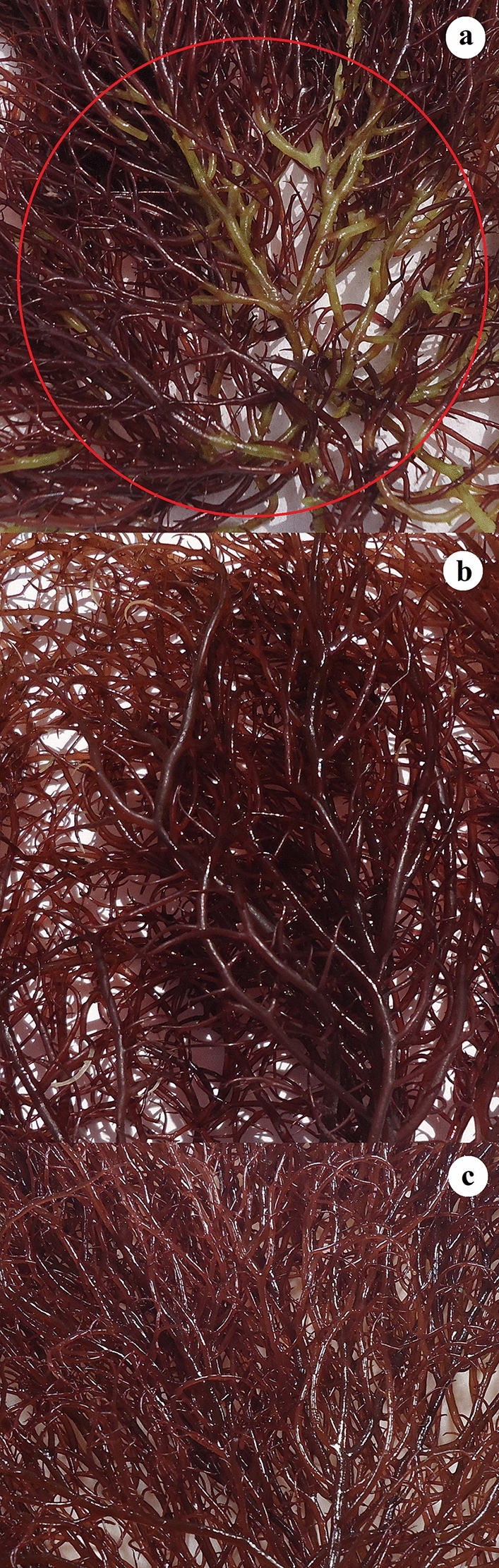
Effects of sulfur on thallus color of *Gracilaria gracilis* after three weeks culture. **(a)** sulfur starved seawater (SSS), **(b) **seawater (SW), and **(c) **sulfur enriched seawater (SES). Yellow circular in Fig. 2a indicate diagnosed bleaching phenomenon known as chlorosis; pigmentation losses of the thalli. Sulfate concentrations [SO_4_^2−^] of the treatments were 2.5 mM, 27 mM and 50 mM for SSS, SW, and SES, respectively.

**Figure 3:**
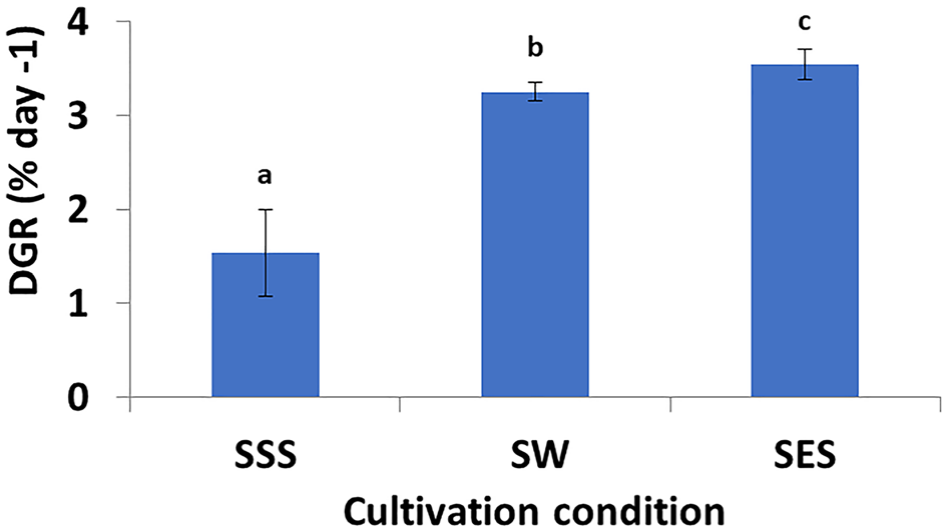
Daily growth rate (DGR, % day^−1^) of *Gracilaria gracilis* expressed as average ± standard deviation (SD).**(a)**Sulfur starved seawater (SSS), **(b)**seawater (SW), and **(c) **sulfur enriched seawater (SES). Sulfate concentrations [SO_4_^2−^] of the treatments were 2.5 mM, 27 mM and 50 mM for SSS, SW, and SES, respectively; (n = 3).

**Figure 4:**
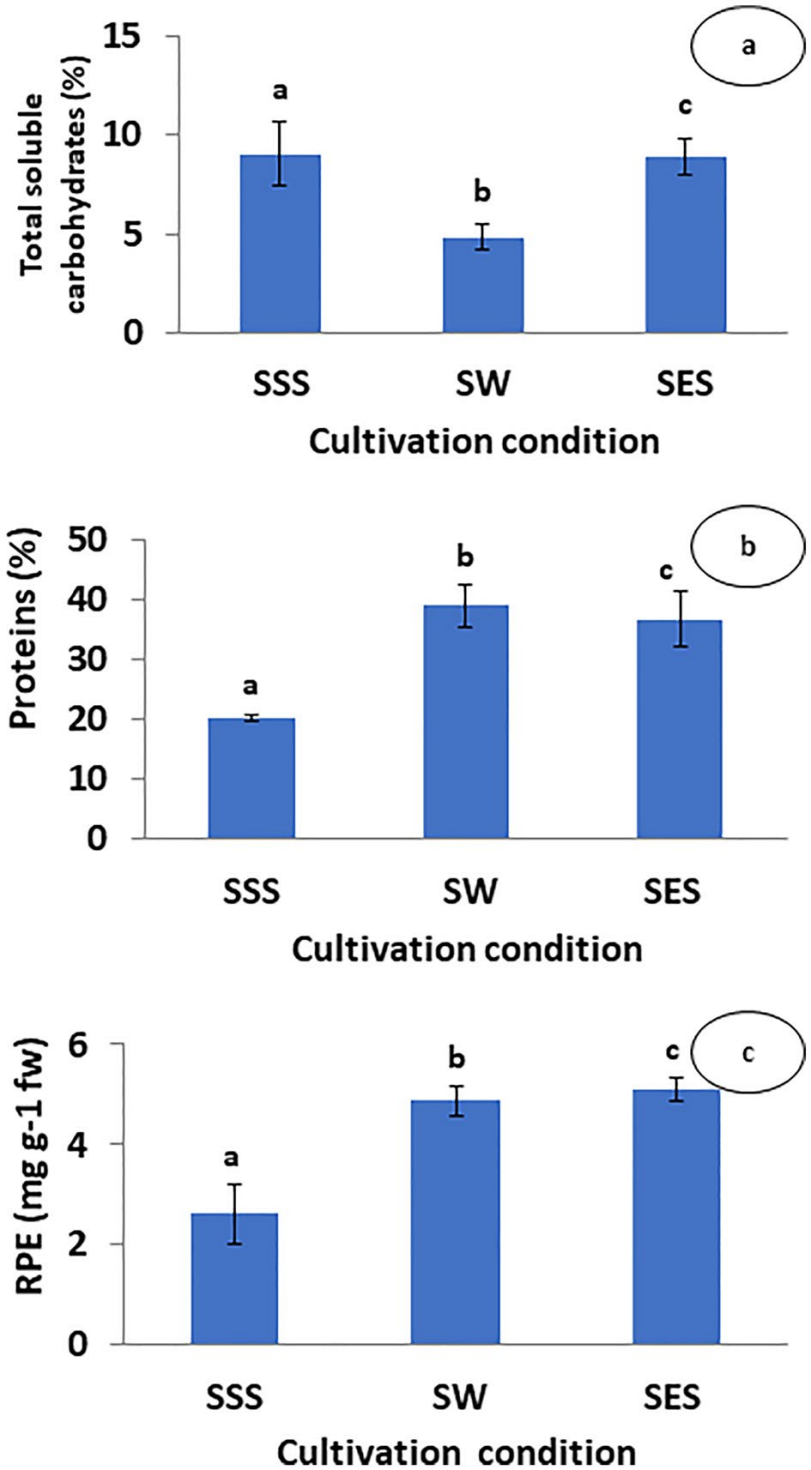
(**a**) Total soluble carbohydrate (%), (**b**) proteins (%) and (**c**) R-phycoerythrin (RPE; mg g^−1^ fw^−1^) contents of *Gracilaria gracilis e*xpressed as average ± standard deviation (SD). Sulfur starved seawater (SSS), seawater (SW), and sulfur enriched seawater (SES). Sulfate concentrations [SO_4_^2−^] of the treatments were 2.5 mM, 27 mM and 50 mM for SSS, SW, and SES, respectively; (n = 9).

The growth pattern of *G. gracilis* was monitored during three weeks of cultivation after sulfur depletion and enrichment experiment. The sampling was performed at the end of experimental period. The thalli grown in SW and SES did not display any color loss, and their color remained consistent (Fig. 2); they continued to grow normally. The thalli subjected to continuous sulfur starvation began to bleach after 15 days and remained in a stationary phase, with no apparent growth. They contained fewer branching than those of other treatments. Finally, thalli lost their rigidity and pigmentation after three weeks (Fig. 2). Observations using a light microscope revealed that depigmentation started with the basal part of the thalli (Fig. 2). Thalli from sulfur enriched seaweater (SES) exhibited a typical shape with well pigmented thalli as those obtained from seawater. The *G. gracilis* daily growth rate (DGR) was affected significantly (One-way ANOVA, *p* < 0.05) by seawater sulfur starvation and enrichment (Fig. 3).The DGR was relatively higher in SW (3.25 ± 0.10% day^−1^) and SES (3.55 ± 0.15% day^−1^) compared to SSS treatment (1.54 ± 0.46% day^−1^), which prove to be the lowest one (Fig. 3). Alga samples from each treatment were hydrolyzed with two enzymes (cellulase and xylanase), and the resulting extracts were analyzed for soluble carbohydrates, proteins and R-phycoerythrin contents. The soluble polysaccharide of *Gracilaria gracilis*, obtained by extraction temperature of 50 °C, is a non-gelling polysaccharide accounting to 9.07 ± 1.61%, 8.92 ± 0.94% and 4.48 ± 0.68% for SSS, SES and SW, respectively. Differences between treatments were significant (p < 0.05). Results indicated that the soluble carbohydrates yield significantly decreased with decreased sulfate concentration in the medium, rather than with its increase compared to that of seawater. The proteins and RPE contents during complete cultivation studies are displayed in Fig. 4. The presented data stand for the mean values for three different experiments with error bars. Examination of photosynthetic pigment RPE and proteins content (Fig. 4) demonstrated treatments effects (p < 0.05). The RPE and protein concentrations increased with the treatment of higher sulfate concentration and decreased with sulfur starvation. Protein and RPE concentrations from sulfur starvation thalli (20.23 ± 0.49% and 2.63 ± 0.59 mg g^−1^ fw respectively) were both approximately 87% and 47% lower than those of seawater and sulfur enriched seawater. In contrast, the difference between proteins and RPE contents of SW compared to SES did not exceed 6% (38.94 ± 3.53% vs 36.61 ± 4.65%) and 5% (5.10 ± 0.244 mg g^−1^ fw vs 4.88 ± 0.30 mg g^−1^ fw).

**Figure 5:**
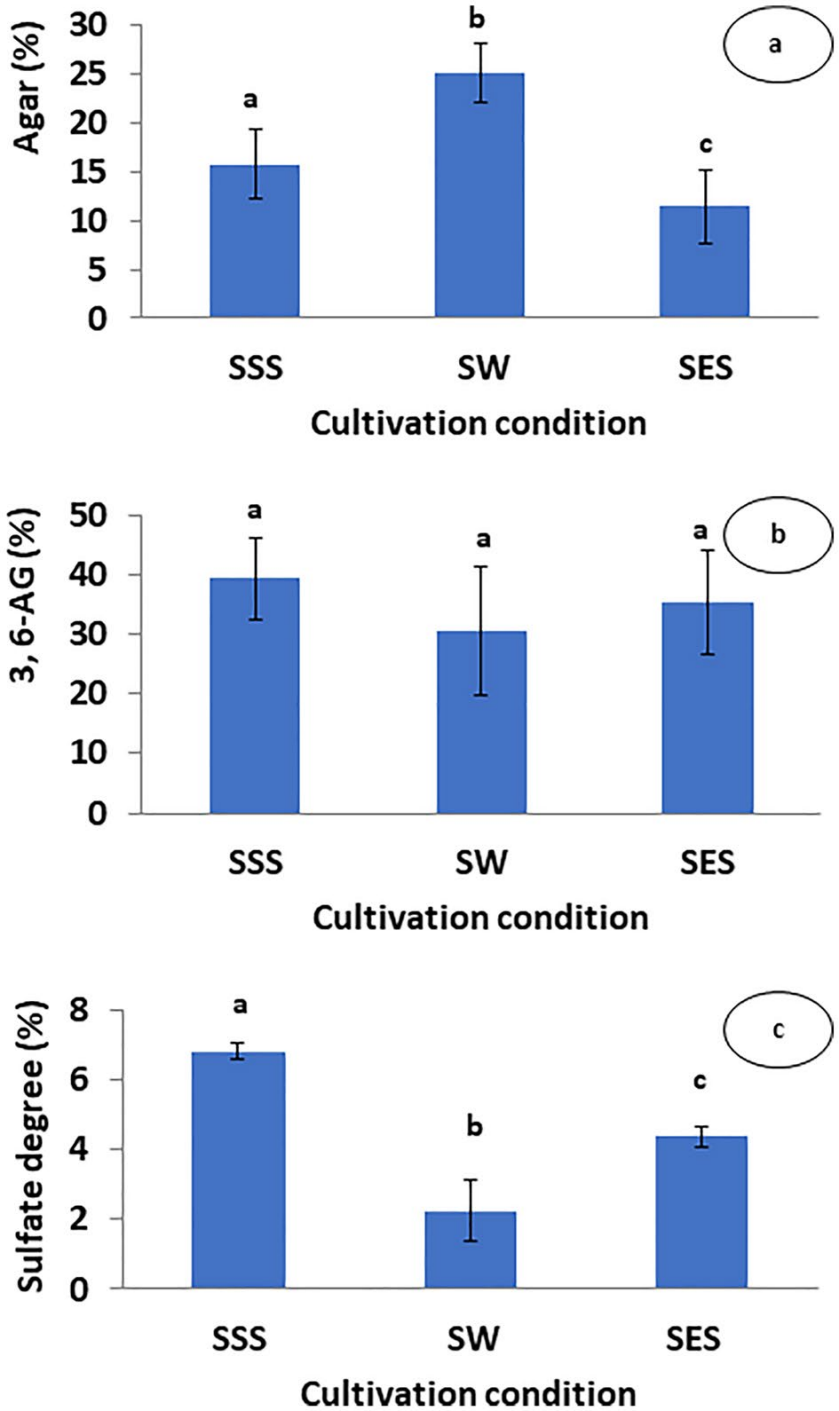
(**a**) Agar (%), (**b**) 3,6-anhydrogalactose content (3,6-AG; %), and (**c**) Sulfate degree (%) of *Gracilaria gracilis* expressed as average ± standard deviation (SD). Sulfur starved seawater (SSS), seawater (SW), and sulfur enriched seawater (SES). Sulfate concentrations [SO_4_^2−^] of the treatments were 2.5 mM, 27 mM and 50 mM for SSS, SW, and SES, respectively; (n = 9).

The cell wall polymer of *Gracilaria *spp. which supplies more than 50% of the world agar is heavily sulfated. Since sulfation reduces the agar quality, it is interesting to investigate the effects of seawater sulfate deprivation and enrichment on the agar yields and composition (sulfate and anhydrogalactose contents). We were interested to find out whether the sulfatation level of agar varies when the sulfate content in seawater varies (to reflect their behavior in the lagoon). Marine environment has stable sulfate content since 40–50 million years ago. The production of sulfated cell wall polysaccharides (such as agar) could be an adaptation strategy that many seaweeds acquired over geological times so as to avoid sulfate toxicity in an environment with high, unchanging sulfate. However, by simulating the conditions of the lagoon, our data revealed that seawater sulfate disturbance (deprivation or enrichment) reduces agar yields and alter its composition. Agar yield significantly affected by treatments were 25 ± 3.5%, 15.71 ± 3.01%, and 11.43 ± 3.75% for SW, SSS, and SES, respectively (Fig. 5). Compared to seawater treatment, agar yield was 37% and 50% higher than that of sulfur starvation and enrichment treatments, respectively. The effects of treatment on the sulfate content in the agar samples were statistically significant (p < 0.001).These findings were indicative that the sulfation of agar may vary according to the sulfate content in the marine environment. However, sulfate degree was 6.83 ± 0.23%, 4.38 ± 0.27% and 2.25 ± 0.9% for seawater sulfur starvation (SSS), enrichment (SES) and seawater (SW), respectively. The sulfate content of SSS and SES were threefold and twofold higher than that of SW samples. It is worth noting that changes in the content of 3,6-anhydrogalactose after sulfate deprivation and enrichment proved to be not inconsistent with the treatments (30.52 ± 10.71%, 39.29 ± 6.75%, and 35.15 ± 8.75% for SW, SSS, and SES, respectively) and differences were not statistically significant (p > 0.05).

## Discussion

Natural seawater has been invested in this research work as it provides all the essential minerals and trace elements, which are difficult to obtain in artificial seawater^39^. Furthermore, prepared seawater is generally used for special applications or when natural seawater is turbid, which was not the case in this study^40^. Given that the natural seawater quality varies seasonally, several artificial mediums were prepared to provide suitable growth conditions for each algal species^41^. In addition, in order to simulate algae growth and the field biochemical composition, it was important to use natural seawater, enriched with artificial medium instead of artificial seawater. Thus, the natural seawater used in this study was enriched with Von Stoch medium for different treatments (SW, SSS, and SES). Yet, the problem arises when we study the seawater nutrients effects on algae growth and their composition. In fact, it was difficult to manipulate sulfate concentrations in the studied sample without seawater properties modifications. Within this framework, numerous authors suggested that an artificial seawater was necessary, to determine the best source of mixed salts^39^.

This research paper center around the turbidimetric and nephelometric methods in order to determine the seawater sulfate concentration. Actually, there are multiple methods, which were used to measure sulfate in water, but in our study we opt for the gravimetric sulfate determination method (the older method) instead of the turbidimetric and nephelometric methods owing to the fact that they are insensitive and tedious. According to^29,38^, this method is based on the precipitation of sulfate with barium and the resulting suspension was monitored photometricly. As a result, it is interesting to use the turbidimetric method with improvements in order to determine sulfate ions within the reach of labs, which do not dispose of sophisticated equipment and to reduce time consumption compared to other ones^42–45^. The American Public Health Association^46^ developed a turbidimetric approach for determining sulfate ions, which has been later updated^29^. The modification process, referring to^29^, has three key goals, reducing the final volume of the reaction (from 105 to 2 mL) and eliminating the 4 min before reading the absorption. Finally, obtaining a linear result over a wider range of sulfate, from 0 to 2 mM, compared to the standard method, which was linear up to 0.4 Mm. In the current study, only a linearity up to 0.2 mM was measured and the result differed from those of^29,46^. Therefore, fitting the data to a second-degree model up to 0.5 mM yields R^2^ = 0.98, but adjusting the data to a linear model yields R^2^ = 0.88. As a result, the difference in values is 10%. Moreover, since the answer y (absorbance of the solution) is positive, the second order model equation: y = 0.5137x^2^ + 0.0197 × has two solutions, one of which is positive, and the other one is negative. The results show that the linear form up to 0.4 mM significantly underestimates the amount of sulfate in all treatments, with a difference exceeding 20%. Relying upon our findings, we invested the linear equation for sulfate determination up to 0.2 mM as a calibration curve. However, for all the studied samples concentrations greater than 0.2 mM, it should be dilute in order to use the linear calibration graph.

Plant cells that are growing in a nutrient-limiting environment increase the acquisition of the limiting nutrient and/or change the flux rates, and intermediate storage options. In addition to the specific responses, more general responses elicited by a variety of stress conditions were obtained. From this perspective, we were basically interested in the following responses in our research: daily growth rate, proteins, RPE, agar, and higher soluble carbohydrate contents. On the other side, Wanner et al.^47^ suggested that this may be a general answer. We judge that they are the most plausible because they give us a deeper insight on how alga biomass will be used in the future.

The DGR of *G. gracilis* cultured in SW and SES was higher than that cultured in SSS. Our finding is consistent with those reported by^18^, who found that the DGR in the indoor *G. gracilis* culture varies from 3% day^−1^ to 5% day^−1^. In outer door culture, these results were lower than those obtained by^48^ in Namibia (5.7% day^−1^to 12.1% day^−1^) and higher than those recorded by^49^ in Tunisia (1% to 1.5%). Thus, the key variations in findings can be attributed to differences in water temperature, light intensity, salinity, and nitrogen concentration^50^. It is possible that the lower DGR of *G. gracilis* obtained after 20 days in SSS refers to the sulfate concentration, which is the only variable among treatments. The sulfur stress triggers extreme stunting of algae and higher plants^51–54^. The red alga *G. gracilis* DGR, on the other hand, was not affected by a five-day sulfate deficiency, as highlighted by^20^. Our findings indicated that if we keep *G. gracilis* cultures under sulfate deprivation for longer than five days, as we did in our case study (20 days), alga growth would decline. The treatments, which reflected not only the conditions of the lagoon but also a long geological period during which the concentration of sulfate ions in the oceans increased, to stabilized at 27 mM (actual value) as indicated by^55^. The red alga *Bangiomorpha pubescens* (first red alga reported), which is quite similar to modern Bangia species, was discovered in the 1,200 Ma (the last period of the Mesoproterozoic). According to^56^ marine sulfate concentrations in the Proterozoic (Paleoprotozoic, Mezoprotezoroic, and Neoproterozoic) remained below 2 mM until about 1.3 Gyr ago, increased to 4.5 mM by 1.2 Gyr ago, and probably reached 7–10 mM by the mid Neoproterozoic. Notably, the presence of red algae, like *Gracilaria*, may have started in waters with sulfate concentrations higher than 4.5 mM. Our findings are supportive for these hypotheses, indicating that *G. gracilis* cannot grow in a medium with a sulfate concentration lower than 2 mM.

The *G. gracilis* protein contents (> 30%) obtained in SW and SES are higher than those previously obtained by^49^ in Bizerte Lagoon and Bizerte Bay (22%-30%). The difference between these results may be assigned to the insufficient nitrogenous concentration in the Lagoon and Bay for *G. gracilis* to produce protein contents higher than 30%. Furthermore, the higher protein content was achieved at salinizes less than 30 psu. The similar proteins contents obtained in both treatments (SW and SES) were attributed to the absence of a negative effect of sulfate on proteins synthesis up to 50 mM. In contrast, the protein content obtained in SSS is lower than those reported in seawater culture. The essential photosynthetic pigments in *G. gracilis* and red algae in general, phycobiliproteins and chlorophyll-a (chla), are depleted during both nitrogen and sulfur deprivation^47^. Their abundance, on the other side, may be a significant physiological parameter in terms of evaluating environmental conditions^57^. R-phycobiliproten, is one of the phycobiliproteins that accumulates in both SW and SES, but at a lower level in SSS. The presence of more RPE in SES indicates that higher sulfate concentrations do not cause algal stress. Under stressful conditions alga uses this pigment for protein synthesis to prevent harmful consequences. Under sulfur deficiency, phycobilisome degradation induces a thalli color shift (Fig. 2) from normal to yellow-green as clarified by^57^. The bleaching process (chlorosis) is similar to that of algae deprived of nitrogen, but phosphate starvation according to several researchers bleached differently. The same bleaching mechanism confirms that depriving algae of nitrogen has the same effect as sulfur deprivation. In this context as^58^ point out, sulfur deficiency has an indirect impact by reducing nitrogen uptake. However, determining the exact sulfur starvation conditions under which *G. gracilis* photosynthesis was drastically reduced, will be extremely significant.

The alga grown under all treatments continues to produce agar, but the sulfate starvation affects its yield indicating that sulfate concentration is relevant. Regarding the agar yields in SSS (15.71 ± 3.01%) and SES (11.43 ± 3.75%), the results obtained were comparable to those reported for *G. gracilis* in Bizerte lagoon^18,49,59^. In SW culture, agar yields (25 ± 3.5%) are 10% higher than those obtained by^49^, in open sea (Bizerte Bay). Friedlander^19^ reported a significant reduction in agar yield after three weeks of sulfate starvation of *Gracilaria conferta* culture, which goes in good conformity with our findings; but are inconsistent with those of^20^ for the same species, over only a five-day culture period. The difference in results may be interpreted in terms of the culture duration, which is insufficient to affect algal growth and agar synthesis. The agar quality, depend mainly on the species, seasons, physiological factors environmental conditions, and the algae's life cycle^60–62^. Only environmental conditions (sulfate concentration) that vary between treatments are included in this analysis. Agar is composed of two main components: agarose and agaropectin, which have different gel strengths^63^. The amount of 3,6-AG and the degree of sulfate have an effect on the physicochemical and rheological properties of agar. Soft gels are generated by agar with a higher sulfate ester and lower 3,6-AG groups, but higher gel consistency is produced by agar with lower sulfates and higher 3,6-AG groups^64^. In our study, agar composition (sulfate degree and 3,6-AG content) obtained in the SW goes in good agreement with that of^49^ obtained in Bizerte Bay (North of Tunisia), which proved to be similar to that of agarose (48% of 3,6-AG and 2% of sulfate). In contrast, due to the higher sulfate degree, the agar obtained in SSS and SES is of lower quality than that recorded in SW. Similar results are obtained in the lagoon by^49^. In contrast, the amount of 3,6-AG found in the SSS and SES is higher than that found in the Bizerte Lagoon as proven by^49,59^. The difference between results may be assigned to sulfate concentration. It is noteworthy that, these studies were under taken in spring, where sulfate concentration is higher than that of SSS (2.5 mM) but lower than that of SES (50 mM). As previously stated, *G. gracilis* produces more soluble carbohydrates (Floridean starch) under SSS and SES, which affects agar synthesis. It seems that at high sulfate concentration (SES treatment), which enhances growth, the metabolism is oriented more towards the production of total soluble carbohydrate than to the agar synthesis as clarified by^65^. Under these two conditions (SSS and SES), the algae mainly produce a non-mature sulfated agar with a low molar ratio and a low sulfated degree, which goes in contrast with anhydrogalactose production. According to^19,66^ an appropriate endogenous sulfate pool in the cell can be recycled from the degradation of proteins such as phycoerythrin. The higher rate of the degradation of these compounds, as depicted by the alga pigmentation loses (Fig. 2) and the RPE content previously mentioned, could account for the higher sulfate degree obtained in SSS treatment compared to SES treatment. Our findings indicate that alga transition from sulfur-depleted to sulfur-enriched seawater need to be explored further in the future for alga culture and exploitation.

## Conclusion

In conclusion, *G. gracilis* was grown under various sulfate concentrations (2.5 mM, 27 mM, and 50 mM), indicating that has the ability to control sulfur acquisition and metabolism in order to sustain its development. The response of alga to sulfate deficiency and enrichment was different as compared to seawater treatment (SW). The sulfur enriched seawater (SES) and seawater sulfur starvation (SSS) treatments exhibited a negative impact on DGR, proteins, RPE, agar yields, and sulfate degree, but no effect on 3,6-AG content. In contrast, the SSS treatment influences positively the *G. gracilis* soluble carbohydrates content. It appears that under sulfur-depleted seawater (SSS), *G.gracilis* tends to be more stressed than the one cultured under sulfur-rich seawater (SES). Owing to the higher agar yield and the better quality obtained, *G. gracilis* cultured in SW proved to be much more interesting than that cultured in SES treatment, despite having similar DGR, proteins, and RPE content. The red alga *G. gracili*s can be ideally cultured in coastal zone with higher nitrogen level. However, outside this area, it can be also cultured in lagoons, whose sulfate content varies from 2.5 mM to 50 mM. At this stage of analysis, we asset that, alga biomass obtained under these conditions will be exploited for their richness on molecules others than agar. Considering all the studied responses, we can conclude that under sulfur deficiency, the carbohydrate and protein fraction were strongly affected, which allowed alga to survive for only three weeks, subsequently, it lost weight and died.

## Data Availability

The data and material used in this study can be provided by the authors after requested.
